# Utilizing questionnaires for medication counselling of patients taking antipsychotics during the COVID-19 pandemic: a single site, community pharmacy-based survey study

**DOI:** 10.1186/s40780-022-00263-w

**Published:** 2022-12-07

**Authors:** Masaki Maehara, Masayasu Sugiyama

**Affiliations:** Sugiyama Pharmacy Co., Ltd, 525-2 Mitsusawa, Ogoori, Fukuoka, 838-0106 Japan

**Keywords:** Community pharmacy, Medication counselling, COVID-19

## Abstract

**Background:**

During the coronavirus disease (COVID-19) pandemic, general strategies for preventing infectious diseases, such as social distancing and the use of protective equipment have resulted in communication barriers between pharmacists and patients in community pharmacies.

**Methods:**

To resolve these communication challenges to medication counselling during the COVID-19 pandemic, during their waiting time at our community pharmacy, we administered two questionnaires to patients receiving at least one antipsychotic drug. The first questionnaire, Questionnaire (A), included questions about any problems with wearing a mask and face shield, forgetting to take medication and adverse effects of their medication. The second questionnaire, Questionnaire (B), included questions regarding the evaluation of medication counselling and the ease of using the first questionnaire.

**Results:**

Questionnaire (A) showed that 26.8% of participants had communication problems due to the mask and face shield and 33.8% sometimes forgot to take their medication. The most common adverse effects of the medications were weight gain (43.7%), dry mouth (39.4%) and sexual dysfunction (31%). In the case of Questionnaire (B), more than 80% responded that it was either very easy or easy to fill out Questionnaire (A). Additionally, 93% participants responded that they felt either very good or good about the pharmacist’s medication counselling using Questionnaire (A).

**Conclusions:**

These results strongly suggest that the utilization of questionnaires in medication counselling may be a useful strategy for preventing communication problems between pharmacists and patients receiving antipsychotics in community pharmacies during and after the COVID-19 pandemic.

## Background

Community pharmacists in Japan are known to be at the forefront of medical services due to their role in dispensing medicine, providing medication instructions and counselling, detecting drug-related problems and responding to patient questions [1]. The interventions of community pharmacies are also needed to prevent infectious diseases, such as for the rational distribution of products (e.g., masks, over-the-counter drugs and prescribed medication), education for appropriate hygiene strategies/warnings against misinformation and support for emotional problems [2, 3].

Coronavirus disease 2019 (COVID-19) is an infectious disease caused by the SARS-CoV-2 virus and it is generally accepted that it spreads through droplets of contaminated fluids and contact transmission. Thus, it is important to implement general strategies utilized to prevent transmission of infectious diseases, such as social distancing and wearing masks in public places. Furthermore, the World Health Organization (WHO) recommends using physical barriers (e.g., sneeze guards) to reduce exposure to COVID-19. Even as we transition toward the end of the pandemic, community pharmacies in Japan need to install acrylic or plastic sneeze guards between patients and pharmacists, who also wear personal protective equipment (PPE), such as masks, face shields or both [4, 5].

However, these basic infection prevention strategies used in community pharmacies may result in communication barriers between patients and pharmacists with respect to medication counselling or instructions, face-to-face communication and medication review. For instance, PPEs have induced a lack of communication between patients and pharmacists in European countries [6]. In addition, a previous study reported a decrease in patients’ opportunities to ask pharmacists questions about their medication owing to a more distant and less accessible setting [7].

Community pharmacists play an important role in the management of patients with problems regarding their mental health and medication, such as the disruption of treatment, barriers to accessing medicine, the risk of relapse, the use of inappropriate drugs and poor adherence. During the COVID-19 pandemic, patients with mental illness, in particular, may have experienced negative effects to their mental health and medication treatment [8]. Community pharmacists educate patients, particularly those with mental illness, about the importance of medication adherence and self-monitoring of the effectiveness and safety of their current medication [8]. Therefore, it is particularly important that communication problems between pharmacists and patients—particularly those with mental illness—in community pharmacies be addressed and resolved during the COVID-19 pandemic.

Here, in order to resolve these communication challenges related to medication counselling during the COVID-19 pandemic, two questionnaires were administered to patients who received at least one antipsychotic drug in our community pharmacy in Japan. The first questionnaire was administered during their waiting time and included questions on problems with wearing PPE, forgetting to take medicines and adverse effects of their medication. The second questionnaire was administered after medication counselling and included questions on the evaluation of the medication counselling, as well as participants’ experiences in responding to the first questionnaire.

## Methods

**Fig. 1:**
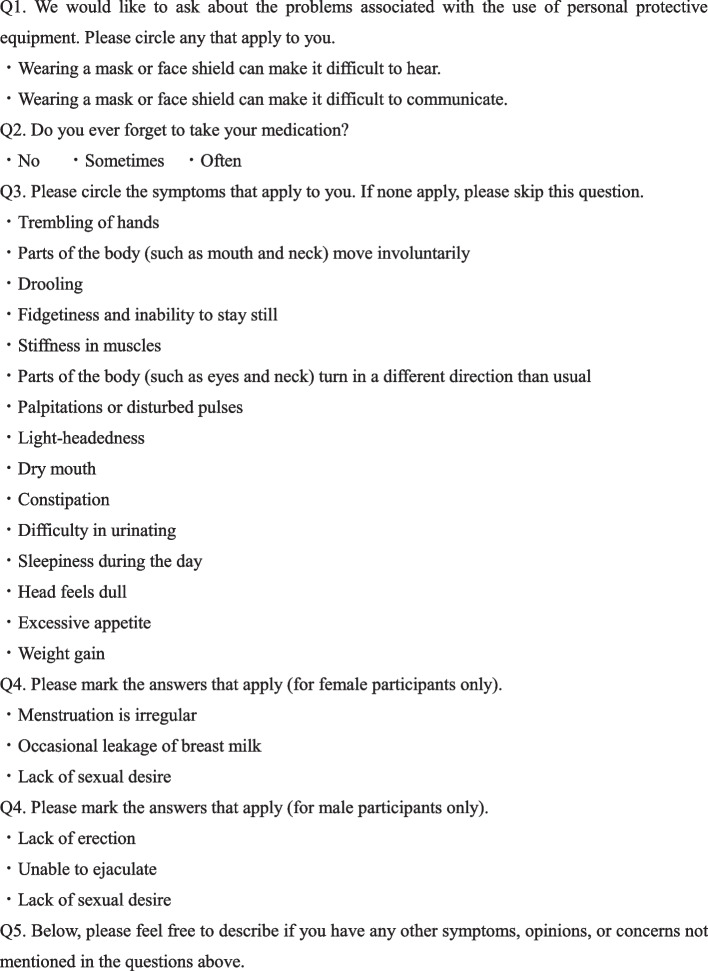
Questionnaire (A)

**Fig. 2:**
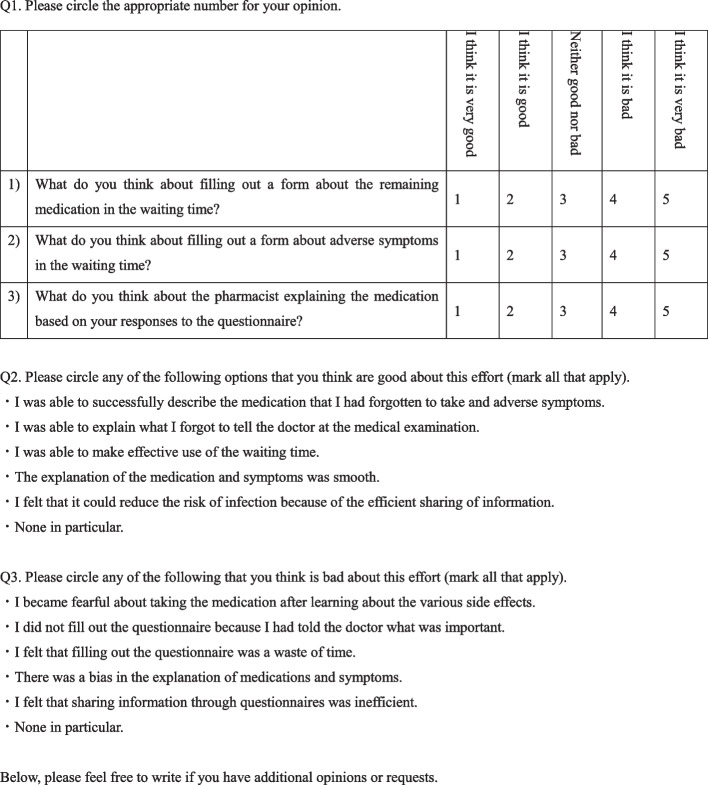
Questionnaire (B)

The study was conducted in our pharmacy located near a psychiatric hospital in Japan from 1 July, 2021, to 31 August, 2021. The participants were between 18 and 64 years of age and were receiving at least one antipsychotic drug at the time. Patient representatives who were obtaining prescribed medication were excluded from the study. In our pharmacy, all participants were advised of infection prevention strategies, such as social distancing and using sanitizers. We administered two printed questionnaires, (A) and (B) (Figs. 1, 2). Informed consent was obtained after the pharmacists explained the aims and procedure of the study, anticipated benefits, withdrawal of consent for the study, personal information protection and contact information. The participants answered Questionnaire (A), which they submitted directly to the pharmacists before medication counselling. A community pharmacist performed medication counselling based on their answers to Questionnaire (A) at the counter, which was separated from other waiting patients by a partition. The participants answered Questionnaire (B) after medication counselling and placed the answered questionnaire in a designated box in the pharmacy. The questionnaire did not require their names, thereby ensuring their anonymity. If the patients agreed to share information observed by questionnaire (A) with the prescribed doctor, the pharmacist provided feedback to doctors by submitting a brief report that summarized information from the questionnaire.

### Contents of Questionnaires (A) and (B)

Questionnaire (A) primarily included questions on problems with wearing a mask and face shield in the pharmacy (Q1), forgetting to take medication (Q2), and to assess medication adherence, and any adverse effects (Q3 and Q4) attributed to the side effects of the antipsychotics, based on previous studies [9–11]. Questionnaire (B) included questions on the evaluation of the pharmacists’ medication counselling, based on the responses from the first questionnaire. The present study was the first to administer Questionnaires (A) and (B) without any pilot tests or associated content modifications.

### Data collection and analysis

Questionnaires (A) and (B) were collected from patients directly and placed in a designated box in our pharmacy, respectively. Also, participant characteristics, such as sex, age, duration of antipsychotics and medication history were obtained from their medication records in our pharmacy.

The participants could choose the appropriate answer(s) for each question and space was allotted for any additional information and comments. The scores for the answers to both questionnaires were calculated and each score was analysed. The difference between the ratio of patients with and without communication problems due to PPE, evaluated using questionnaire (A), was analysed by Fisher’s exact test (the statistically significant *p*-value was < 0.05). All analyses were performed with EZR version 1.52 (Saitama Medical Centre, Jichi Medical University, Saitama, Japan); all answers to open-ended questions were categorized as a positive or negative opinion and summarized by the authors. This study was reviewed by the Fukuoka Pharmaceutical Association Academic Ethics Review Committee (approval number; 2021–001 and 2021–001-revison-1).

## Results

### Participant characteristics

As shown in Table 1, 71 patients participated in the study by responding to two printed questionnaires: (A) and (B). In total, 73 patients were asked to participate, but 2 patients declined due to (i) unwillingness to write and (ii) insufficient time to participate. Overall, 63.4% of the participants were women, and the average age of the participants was 42.4 years. All participants received at least one antipsychotic drug from our pharmacy; 83.1% of participants were on second-generation antipsychotics, 31% were on first-generation antipsychotics and 38% were administered combined antipsychotics. The durations of antipsychotic use were within 180 days (9.9%), from 180 to 365 days (5.6%) and over 365 days (78.9%). In addition, some participants were also taking hypnotics (73.2%), antidepressants (42.3%), mood stabilizers (22.5%), anticholinergic drugs (16.9%) and psychostimulants for attention deficit hyperactivity disorder (ADHD) (4.2%).

**Table 1:** Patient characteristics

Characteristics	Patient-numbers (% of total (71) participants)
(A) Sex	
Women	45 (63.4%)
Men	26 (36.6%)
(B) Age	
Mean Value	42.4 (range 21–63)
Standard deviation	11.4
(C) Duration of taking antipsychotics	
< 180 days	7 (9.9%)
180–365 days	4 (5.6%)
> 365 days	56 (78.9%)
Unknown^a^	4 (5.6%)
(D) Drugs	
Second generation antipsychotics (SGA)	59 (83.1%)
First generation antipsychotics (FGA)	22 (31.0%)
Combined administration of antipsychotics	27 (38.0%)
BZP/Orexin receptor blockers/ramelteon	52 (73.2%)
Antidepressants	30 (42.3%)
Mood stabilizers	16 (22.5%)
Anticholinergic drugs	12 (16.9%)
Psychostimulants	3 (4.2%)

^a^Due to insufficient information about medication before coming to our pharmacy, the duration in which medication was being taken is unclear

### Questionnaire (A): problems of wearing a mask and face shield, forgetting to take medicines and adverse effects of medications

In the present study, all pharmacy staff and patients in the community pharmacy used PPE, such as masks or face shields, and there were plastic sneeze guards between the patients and pharmacists. As shown in Table 2 (Q1), 26.8% of participants responded that these basic strategies (using a mask or face shield to prevent COVID-19) resulted in communication barriers, particularly, problems with hearing (18.3%) and speaking (14.1%). Interestingly, 73.2% of the patients did not report having communication problems owing to PPE.

**Table 2:** Responses to Questionnaire (A)

Questions	Choice	Number	% of total (71) participants
Q1. Patients with problems caused by a mask or face shield (at least one)		19	26.8
Wearing a mask or face shield can make it difficult to hear		13	18.3
Wearing a mask or face shield can make it difficult to communicate		10	14.1
Q2. Do you ever forget to take your medication?	No	47	66.2
	Sometimes	24	33.8
	Often	0	0
Q3. Patients with the symptoms mentioned below (at least one)		62	87.3
Weight gain		31	43.7
Dry mouth		28	39.4
Sleepiness during the day		17	23.9
Excessive appetite		17	23.9
Constipation		16	22.5
Light-headedness		15	21.1
Feeling dull		14	19.7
Trembling of hands		13	18.3
Fidgetiness and inability to stay still		8	11.3
Palpitations or disturbed pulses		8	11.3
Difficulty in urinating		5	7.0
Stiffness in muscles		4	5.6
The body moves involuntarily		2	2.8
The body turns in a different direction than usual		2	2.8
Drooling		2	2.8
Q4. Patients with sexual dysfunction (at least one)		22	31.0
Female participants with sexual dysfunction (at least one)		13	28.9^a^
Menstruation is irregular		8	17.8^a^
Lack of sexual desire		5	11.1^a^
Occasional leakage of breast milk		1	2.2^a^
Male participants with sexual dysfunction (at least one)		9	34.6^b^
Lack of sexual desire		7	26.9^b^
Unable to ejaculate		4	15.4^b^
Lack of erection		1	3.8^b^

^a^Total number of female participants (*n* = 45)

^b^Total number of male participants (*n* = 26)

Table 2 (Q2) also shows that 66.2% of the participants did not forget to take medication, while 33.8% forgot to do so sometimes and no patients forgot to take medications often. Regarding the adverse effects of medications (Table 2, Q3), 87.3% experienced at least one adverse effect. Common adverse effects were weight gain (43.7%), dry mouth (39.4%), sleepiness during the day (23.9%), increased appetite (23.9%), constipation (22.5%), light-headedness (21.1%), feeling dull (19.7%), trembling hands (18.3%), fidgetiness and inability to stay still (11.3%) and palpitations or disturbed pulses (11.3%).

**Table 3:** Responses to Questionnaire (B) – evaluation questions (before medication counselling)

Question number	Question contents	Choice	Number	% of total participants (71)
1	What do you think about filling out a form about forgetting to take medicines, during your waiting time?	Very good	24	33.8
		Good	33	46.5
		Neutral	14	19.7
		Bad	0	0
		Very bad	0	0
2	What do you think about filling out a form about the adverse symptoms you are experiencing during your waiting time?	Very good	31	43.7
		Good	29	40.8
		Neutral	11	15.5
		Bad	0	0
		Very bad	0	0
3	What do you think about the pharmacist explaining the medication based on your responses to Questionnaire (A)?	Very good	48	67.6
		Good	18	25.4
		Neutral	5	7.0
		Bad	0	0
		Very bad	0	0

In addition, as shown in Table 2 (Q4), 31% of the participants are currently having sexual dysfunction and 28.9% (13/45) of female participants reported sex-related problems, including menstrual disorders, lactation problems and lack of sexual desire. In the case of male participants, 34.6% (9/26) reported lack of sexual desire, erectile disfunction and inability to ejaculate.

### Questionnaire (B): an evaluation of Questionnaire (A)

As shown in Table 3, most participants responded that they felt either very good or good about filling out Questionnaire (A). Participant responses regarding both forgetting to take medication (33.8 + 46.5 = 80.3%) and adverse effects (43.7 + 40.8 = 84.5%) were recorded. Moreover, 93% (67.6 + 25.4 = 93.0%) of the patients evaluated the pharmacist’s medication counselling based on Questionnaire (A) as either very good or good. Table 4 also shows that 87.3% of the participants mentioned at least one advantage of the pharmacist providing medication counselling using Questionnaire (A). The participants highlighted the following advantages: (1) it helped us understand the symptoms and medication (50.7%), (2) it prompted us to report both our forgetting to take medicines, as well as the side effects of the ones we were taking (46.5%), (3) it helped us use our waiting time efficiently (45.1%), (4) it was a reminder that we had forgotten to share something with the doctors (33.8%) and (5) it helped us reduce the risk of infection (12.7%). However, a few participants noted some negatives in the present medication counselling because it enhanced the fear of adverse side effects (2.8%) and because all the information required in Questionnaire (A) was already shared with the prescribing doctor (2.8%).

**Table 4:** Responses to Questionnaire (B) -Advantages and Disadvantages of Questionnaire (A)

[A] Advantages		
Advantages of Questionnaire (A)	Number (n)	Total % of participants, out of 71
Participants who mentioned at least one advantage of the intervention	62	87.3
The explanation of the medication and symptoms was smooth	36	50.7
I was able to successfully describe the medicines that I had forgotten to take and the adverse effects	33	46.5
I was able to make effective use of the waiting time	32	45.1
I was able to explain what I forgot to tell the doctor at the medical examination	24	33.8
I felt that the risk of infection could be reduced because of the efficient sharing of information	9	12.7
None	9	12.7
[B] Disadvantages		
Disadvantages of Questionnaire (A)	Number (n)	Total % of participants out of 71
Participants who mentioned at least one disadvantage of the intervention	4	5.6
I became fearful about taking the medication after learning about the various adverse symptoms	2	2.8
I did not fill out the questionnaire because I had told the doctor what was important	2	2.8
I felt that filling out the questionnaire was a waste of time	0	0
There was a bias in the explanation of the medication and symptoms	0	0
I felt that sharing information through questionnaires was inefficient	0	0
None in particular	67	94.4

Some participants in the study expressed other opinions and questions in the allotted space in Questionnaire (B), which are as follows: “The questionnaire was very helpful because I sometimes forget to mention some symptoms and questions to the prescribing doctor.” “The questionnaire provided me with good opportunities to check my medication problems and to share my information with community pharmacists.” “The pharmacy’s intervention helped me describe some adverse effects; this also proved to be efficient use of the waiting time.” However, some patients made negative comments, such as: “The questionnaire would be uncomfortable for some patients who are unable to write due to diseases such as Parkinsonism.” “I did not want to describe real symptoms such as sensitive adverse events because of other patients in the waiting room.”

### Evaluation of Questionnaire (A) in patients with or without communication problems due to PPE

When compared with the ratio of patients without communication problems due to PPE, the ratio of patients with problems had significantly increased with respect to filling out a form about forgetting to take medication (Table 5, Q1, *p* = 0.0148) and adverse effects of the medication (Table 5, Q2, *p* = 0.0296) during waiting time. On the other hand, there was no significant difference between the ratio of patients with and without problems due to PPE who selected “Very good/good” in response to the question, “What do you think about the pharmacist explaining the medication based on your responses to Questionnaire (A)?” (Table 5, Q3, *p* = 0.315).

**Table 5:** Relationship between communication problems due to PPE and evaluation of medication counselling using Questionnaire (A)

Evaluation of Questionnaire (A)		Ratio (%)	Ratio (%)	*p*-value
		Problems with PPE (*n* = 19)	No problems with PPE (*n* = 52)	
Q1 What do you think about filling out a form about forgetting to take medicines, during your waiting time?	Very good/ Good Neutral	19 (100%) 0	38 (73.1%) 14 (26.9%)	0.0148*
Q2 What do you think about filling out a form about the adverse symptoms you are experiencing during your waiting time?	Very good/Good Neutral	19 (100%) 0	41 (78.8%) 11 (21.2%)	0.0296*
Q3 What do you think about the pharmacist explaining the medication based on your responses to Questionnaire (A)?	Very good/Good Neutral	19 (100%) 0	47 (90.4%) 5 (9.6%)	0.315

Ratio; calculated as the number of patients who selected a certain answer divided by the total number of patients in that group

## Discussion

Our present results strongly suggest that during the COVID-19 pandemic, there was a benefit to using the questionnaire for medication counselling in a community pharmacy to prevent communication problems due to PPE between pharmacists and patients receiving antipsychotics.

A previous cross-sectional study in the Netherlands reported that 55.8% of community pharmacists could not conduct medication reviews during the COVID-19 pandemic [7]. In other studies, one-third of community pharmacists mentioned that general mitigation strategies for COVID-19 such as social distancing caused communication barriers between pharmacists and patients [11]. In the present study, 26.8% of the participants had communication problems with community pharmacists in terms of hearing (18.3%) and communication (14.1%) due to PPE such as masks or face shields (Table 2; Q1). These results suggest that basic strategies such as social distancing and utilizing plastic sneeze guards, as well as the use of PPE to prevent COVID-19, resulted in communication barriers between pharmacists and patients.

It is important for community pharmacists to obtain information about medications that patients have forgotten to take as this may be related to poor and/ or non-adherence to the medication regimen. According to a recent review, satisfactory medication adherence for oral antipsychotics in patients with schizophrenia is approximately 70%, as estimated by measurements taken from electronic adherence monitoring devices [12]. The results of the present study also showed that 66.2% of participants receiving at least one oral antipsychotic drug did not forget to take their medicines, while 33.8% forgot to do so sometimes (Table 2; Q2). During the COVID-19 pandemic, patients with mental illness in particular, may face various problems, including medication non-adherence due to various barriers, such as less communication with healthcare providers [13]. Since 80.3% of the participants taking antipsychotic medications willingly admitted in Questionnaire A to forgetting to take their medication (Table 3), questionnaires may be useful for obtaining information from patients with mental illness about the remaining medication and medication adherence, particularly during situations such as the COVID-19 pandemic.

The most common side effects in patients receiving antipsychotics are reported to be daytime sleepiness, weight gain, sexual dysfunction, akathisia and dry mouth [14]. The present study showed that 87.3% experienced at least one adverse effect and revealed that the most common adverse effects were weight gain, dry mouth, sleepiness during the day, increased appetite, constipation, light-headedness, feeling dull and trembling of hands (Table 2; Q3). Therefore, it is possible that the adverse effects observed in the present study may be partially related to antipsychotic drugs and/or other medications such as hypnotics, antidepressants, mood stabilizers, anticholinergic drugs and psychostimulants for ADHD, although the mechanisms underlying these adverse effects are unclear.

In Japan, the important role of the community pharmacist in checking for side effects and reporting them to the prescribing doctors is well known. Among the side effects caused by antipsychotics, weight gain and sexual dysfunction specifically during the COVID-19 pandemic needs to be noted, because the pandemic changed people’s routine and day to day activities due to self-quarantine, less exercise, increased stress and changes in behaviours. These changes could affect weight gain and lack of weight management in adults with obesity and mental health problems [15, 16]. In addition, previous studies have suggested that the COVID-19 pandemic led to a decrease in sexual activity and satisfaction [17, 18]. In general, patients tend to avoid discussions regarding sensitive problems, such as sexual dysfunction, owing to a weak relationship between patients and physicians [14]. Previous studies have also shown that patients with sexual dysfunction tend not to share their problems directly with the prescribing doctor in Japan [14, 19]. Moreover, details regarding sexual dysfunction are difficult to obtain because of their personal nature. However, in the present study, 84.5% of patients answered either very good or good regarding the ease of filling out Questionnaire (A) with respect to their adverse effects (Table 3), including weight gain and sexual dysfunction. These results imply that the questionnaire is a useful tool for the early detection of adverse effects that may be, in part, related to the side effects caused by medicines including antipsychotics.

Reporting medication problems such as medication adherence, adverse effects and other problems may help in communication and discussion with patients during medication counselling with the pharmacist. In the present study, 93% of the participants had a positive attitude toward the medication counselling using Questionnaire (A) (Table 3), and most of the participants (87.3%) acknowledged the advantages of medication counselling using Questionnaire (A) in terms of sharing information, effective medication counselling and waiting time efficiency (Table 4). In addition, patients with problems due to PPE had a higher likelihood to indicate forgetting to take medication and adverse effects in questionnaire (A) when compared with patients without problems (Table 5). Collectively, these results strongly suggest that the utilization of questionnaire (A) would be one of the prudent strategies to avoid the induction of communication problems caused by PPE between pharmacists and patients with antipsychotics during the COVID-19 pandemic. The present results also showed that 73.2% patients did not mention communication barriers caused by PPE (Table 2; Q1), while more than 73% of patients without problems due to PPE felt either very good or good about our strategy using the questionnaire (Table 5), indicating the usefulness of the questionnaire even beyond the COVID-19 pandemic.

### Limitations

This study has following limitations: (1) Questionnaire (A) did not inquire about the exact number of remaining medications, which may have caused the overestimation of reported remaining medication; (2) a correlation was not detected between adverse effects and medications such as antipsychotics, indicating that adverse effects may be caused not only by the administration of drugs but also by other factors such as lifestyle changes; (3) clinical outcomes, including changes in prescription after feed-back to doctors and the ratio of drug withdrawal, which is associated with adverse effects, and the improvement of these adverse effects by the intervention were not evaluated; (4) the sample size was small (*n* = 71); and (5) it was a single-centre study. Therefore, further studies are needed to clarify the utility of questionnaires for the improvement of clinical outcomes.

## Conclusion

In conclusion, these results strongly suggest that utilizing questionnaires in a community pharmacy during and after the COVID-19 pandemic in Japan may be a very useful strategy to prevent communication problems due to PPE between pharmacists and patients receiving antipsychotics.

## Data Availability

All data generated or analysed during this study are included in this published article.

## Abbreviations

ADHD: Attention deficit hyperactivity disorder
COVID-19: Coronavirus disease 2019
PPE: Personal protective equipment
WHO: World Health Organization
